# Initial evaluation in the climacteric: Number 5 - May 2022

**DOI:** 10.1055/s-0042-1750282

**Published:** 2022-06-13

**Authors:** Luiz Francisco Cintra Baccaro, Lúcia Helena Simões da Costa Paiva, Elizabeth Jeha Nasser, Ana Lúcia Ribeiro Valadares, Célia Regina da Silva, Eliana Aguiar Petri Nahas, Jaime Kulak Junior, Márcio Alexandre Hipólito Rodrigues, Marco Aurélio Albernaz, Maria Celeste Osório Wender, Maria Célia Mendes, Rita de Cassia de Maio Dardes, Rodolfo Strufaldi, Rogerio Cesar Bocardo, Luciano de Melo Pompei

**Affiliations:** Universidade Estadual de Campinas, Campinas, SP, Brazil; Universidade Estadual de Campinas, Campinas, SP, Brazil; Departamento de Ginecologia e Obstetrícia, Faculdade de Medicina do ABC, Santo André, SP, Brazil; Universidade Estadual de Campinas, Campinas, SP, Brazil; Universidade Federal do Rio de Janeiro, Rio de Janeiro, RJ, Brazil; Universidade Estadual Paulista “Júlio de Mesquita Filho”, Botucatu, SP, Brazil; Universidade Federal do Paraná, Curitiba, PR, Brazil; Universidade Federal de Minas Gerais, Belo Horizonte, MG, Brazil; Universidade Federal de Goiás, Goiânia, GO, Brazil; Universidade Federal do Rio Grande do Sul, Porto Alegre, RS, Brazil; Faculdade de Medicina de Ribeirão Preto, Universidade de São Paulo, Ribeirão Preto, São Paulo, Brazil; Departamento de Ginecologia, Escola Paulista de Medicina, Universidade Federal de São Paulo, São Paulo, SP, Brazil; Departamento de Ginecologia e Obstetrícia, Faculdade de Medicina do ABC, Santo André, SP, Brazil; Faculdade de Medicina de Jundiaí, Jundiaí, SP, Brazil; Departamento de Ginecologia e Obstetrícia, Faculdade de Medicina do ABC, Santo André, SP, Brazil

## Key points

- Climacteric syndrome is a set of signs and symptoms resulting from the interaction between sociocultural, psychological and endocrine factors occurring in aging women. Its diagnosis is clinical in women with the expected age group for ovarian hypofunction. The term “menopause” refers to the date of the woman's last menstrual bleeding episode and is defined retrospectively.
- Women in menopausal transition have disease prevention and health promotion needs. Hypoestrogenism associated with aging and metabolic syndrome can lead to lower quality of life and increased occurrence of cardiovascular disease.
- The medical consultation of climacteric women is an excellent opportunity of screening for chronic diseases and neoplasms.
- Complementary evaluation for climacteric women must be carried out judiciously. The benefit and risk of each test must be considered. Complementary tests without a specific definition of a diagnostic and therapeutic plan should be avoided.
- Menopausal hormone therapy (HT) may be indicated to treat climacteric symptoms. Detailed clinical history and rational complementary exams are essential to define the therapeutic plan and follow-up of women undergoing HT.

## Recommendations

- For women over 45 years of age with symptoms suggestive of hypoestrogenism such as typical hot flashes, the diagnosis of climacteric syndrome is clinical. The date of menopause is defined retrospectively, after 12 months of amenorrhea in a woman over 45 years of age. When in doubt, two doses of follicle-stimulating hormone (FSH) 4-6 weeks apart are recommended. Values above 25 mIU/mL indicate the beginning of menopausal transition. For women under 45 years of age who complain of irregular uterine bleeding pattern and infrequent menstrual cycles, the performance of complementary tests for investigation is recommended.
- The diagnosis of genitourinary syndrome of menopause should be made proactively, with questioning directed to urogenital symptoms associated with a thorough gynecological examination.
- In women at usual risk, breast cancer screening should begin at age 40. Mammography is the recommended exam yearly (if normal). In women with dense breasts, ultrasound should be considered as a supplement to mammography. Breast cancer screening can be interrupted when life expectancy is less than seven years or if there are no clinical conditions for diagnosis or treatment.
- Cervical cancer screening is performed using periodic oncotic cytology. After performing two consecutive negative cytology tests at annual intervals, it is recommended to do the exam every three years. Screening can be stopped after age 64 if the patient has two consecutive negative tests in the previous five years. The gynecological physical examination should be part of the periodic gynecological evaluation regardless of the woman no longer having an indication for cervical cancer screening.
- Requesting pelvic and transvaginal ultrasound for women (at usual risk) without signs or symptoms suggestive of disease is not cost-effective. For symptomatic women, such as those with abnormal uterine bleeding, postmenopausal vaginal bleeding, or abdominal discomfort, transvaginal pelvic ultrasound is cost-effective and is the initial complementary test of choice to evaluate uterine and ovarian diseases.
- For women at normal risk and over 50 years of age, colorectal cancer screening is recommended.
- Screening for risk factors for cardiovascular disease, including hypertension, diabetes mellitus, obesity, smoking and dyslipidemia, should always be considered in the consultation of climacteric women. Researching all criteria for the definition of metabolic syndrome is essential. Menopausal transition is a window of vulnerability for the development of mood swings, such as depressive disorder. Identifying women with depressive symptoms is important to institute the appropriate therapy.
- Bone densitometry is recommended for screening for osteoporosis in all women aged 65 years and older. Densitometry is also indicated for climacteric women under 65 years of age who have at least one risk factor for osteoporosis. The FRAX-Brasil, analyzed according to the recommendation of the National Osteoporosis Guideline Group (NOGG), can also be used to assess the need for densitometry.
- Sexually transmitted infections (STIs) during menopause and postmenopause cannot be underestimated. Behavioral counseling and treatment of genitourinary syndrome of menopause are important tools to decrease the risk of STIs. Screening for STIs should be performed based on each patient's clinical history data.
- Thyroid diseases are prevalent in aging women and can increase morbidity and mortality. Clinical evaluation of the thyroid should be performed routinely during physical examination of climacteric women. Women over 60 years of age or with symptoms of thyroid dysfunction should be evaluated initially with a thyroid-stimulating hormone (TSH) measurement.

## Background

The transition between reproductive and non-reproductive stages in a woman's life is called climacteric. 1 At this stage, women have numerous needs for disease prevention and health promotion, and physicians must be aware of a series of conducts aimed at optimizing the quality of life. 2 The gynecological consultation is an excellent opportunity to meet these needs. A review of the pertinent literature on the subject was performed in order to systematize the initial workup during the gynecological consultation of climacteric women. The most relevant results are shown below, divided into: diagnosis of menopause and climacteric syndrome; opportunistic screening of chronic diseases and neoplasms; specific tests aimed at women undergoing menopausal HT.

### How are menopause and climacteric syndrome diagnosed?

Aging leads to progressive ovarian failure, determining the interruption of ovulatory cycles and cessation of menstrual bleeding. With the objective of standardizing the definition of the different stages of reproductive aging, the STRAW system, Stages of Reproductive Aging Workshop, was developed. 3 The characterization of the reproductive period, menopausal transition and postmenopausal period is performed based on patterns of symptoms and laboratory findings.

The date of the woman's last menstrual bleeding episode is defined as menopause. 1 It occurs at 51 years of age, on average, and 90% of women experience menopause between 45 and 55 years of age. 4 Its definition is performed retrospectively after 12 months of amenorrhea in women in the expected age group for the menopausal transition. 4 Premature ovarian failure is a syndrome resulting from the loss of ovarian activity before 40 years of age. 5 This condition affects approximately 1% of women. 6 Menopause between 40 and 45 years of age affects approximately 5% of women and has been called early menopause. 6

The term “climacteric syndrome” refers to the set of symptoms and signs resulting from the interaction between sociocultural, psychological and endocrine factors that occur in aging women. 1 Its diagnosis is based on a detailed anamnesis complemented by a thorough physical examination. 2

Vasomotor symptoms, also known as hot flashes, are the most frequently associated with menopausal transition. They consist of sudden sensations of heat in the central region of the body, most notably in the region of the face, chest and neck, and last an average of three to four minutes. 7 There is often an increase in heart rate, peripheral vasodilation, elevation of skin temperature and sweating. They can be associated with insomnia when occurring at night. 8

Women often seek care due to changes in the menstrual cycle in the menopausal transition. As a consequence of decreased ovarian production of inhibin B at the end of the fourth decade of life, an increase in serum concentrations of FSH and estradiol may occur at the beginning of the cycle, causing a shortening of the follicular phase. Progesterone level in the luteal phase also decreases given the deterioration of the quality of the corpus luteum. One of the first signs of reduced ovarian reserve is the shortening of the interval between menstruations. 9

Over the years, the process of follicular depletion continues and anovulation becomes more and more frequent. Due to the lack of progestational opposition, the interval between menstrual cycles is longer, passing to 40 to 50 days. The increase in the interval between menstrual cycles occurs at 47 years of age, on average. 9 Longer episodes of amenorrhea begin to occur, interrupted by episodes of menstrual bleeding of variable volume. This menstrual bleeding pattern can last from one to three years before menopause. 9

For women over 45 years of age with complaints suggestive of hypoestrogenism, such as vasomotor symptoms and typical changes in menstrual pattern (infrequent uterine bleeding), the diagnosis of climacteric syndrome is clinical and does not require confirmation by other complementary tests. 2 In case of doubts if symptoms are resulting from a drop in ovarian estradiol production, FSH measurement in the early follicular phase can confirm the diagnosis. Values above 25 mIU/mL may indicate the beginning of menopausal transition, although concentrations may have great daily variability during this phase. 9 When necessary, two dosages at four to six weeks interval should be performed. 2 For women under 45 years of age who complain of abnormal uterine bleeding with an irregular pattern and infrequent menstrual cycles, even if the clinical picture is compatible with hypoestrogenism, a complementary evaluation is recommended for investigation of symptoms and exclusion of other causes of menstrual irregularity. 9

### How screening for chronic diseases and neoplasms in the climacteric should be?

During the climacteric, an individual evaluation of each woman is essential to meet her needs for disease prevention and health promotion. 2 Next, details on screening for gynecological malignancies, colorectal cancer, risk factors for cardiovascular disease, osteoporosis, depression and sexually transmitted infections (STIs) are presented.

### How to screen for breast cancer?

Breast cancer is the second most frequent neoplasm among Brazilian women, with an estimated incidence of 66,280 new cases for each year of the 2020-2022 triennium, corresponding to an estimated risk of 61.61 new cases per 100,000 women. 10 The objective of screening is to reduce the need for mutilating procedures and increase survival. 11 Mammographic screening can reduce breast cancer mortality by approximately 20% and reduce the risk of advanced-stage breast tumors in women over 50 years of age. 12 Breast ultrasound should not be used as the only screening method due to the lack of studies in women at normal risk, but it should be used as a complementary method to mammography in women with dense breasts. The use of magnetic resonance imaging is not recommended as a screening method in women at normal risk. 13

Breast cancer screening can present risks such as overdiagnosis, overtreatment, and false-positive results. 14 The shared decision between physician and patient should be considered to define the age of onset, periodicity, and when to stop screening. Febrasgo suggests that breast cancer screening starts at age 40 for women at normal risk. Mammography is the recommended exam yearly (if normal). Breast cancer screening can be interrupted when life expectancy is less than seven years or when there are no clinical conditions for the diagnosis or treatment of a woman with an abnormal test result ( Chart 1 ). 13 The screening in patients at high-risk for breast cancer is outside the scope of this publication.

**Chart 1.** Recommendations for breast cancer screening in women at usual risk

Febrasgo/SBM/CBR	
Clinical examination by a health professional	Recommended
Self-exam	Recommended
Recommended age to start mammography	40 years
Frequency of mammography	Yearly
Recommended age to end mammography screening	Discontinue when life expectancy < 7 years or there are no clinical conditions for diagnosis/treatment of altered exam

### How to screen for cervical cancer?

Cervical cancer is the fourth most frequent neoplasm among Brazilian women, with an estimated 16,590 new cases for each year of the 2020-2022 triennium and an estimated risk of 15.43 cases per 100,000 women. 10 The Brazilian Ministry of Health recommends the Pap smear test as the method of choice for screening for precursor lesions. 15 It is recommended to start collection at 25 years of age for women who have already started sexual activity with an year interval between the first two exams. If the first two results are normal, collection is performed at a three-year interval. If the patient has two consecutive negative tests in the last five years, cytological screening can be interrupted after the age of 64 if the woman has never had a history of pre-invasive precursor lesion, including if there is a change of sexual partner. 15 For women in this age group who have never been screened, two Pap smears at one to three year-intervals are recommended before stopping the screening. 15 Menopause genitourinary syndrome, previously known as urogenital atrophy, 11 can lead to the occurrence of results such as “atypical squamous cells of undetermined significance” (ASC-US) in cytopathological examinations of the cervix. The use of topical estrogen for at least 21 days before the next collection can be useful in these cases. 16 The use of human papillomavirus (HPV) detection tests associated with cytology is recommended by some national and international societies for women from the age of 30 years. As it is more sensitive and has a high negative predictive value, the screening strategy including the HPV test allows increasing the interval between collections from three to five years when both results are negative. 16 Women without a history of precursor cancer lesions of the cervix can stop screening after performing a total hysterectomy for benign disease. 15 It is extremely important to maintain the periodical gynecological clinical evaluation by means of gynecological examination regardless of cytopathological examination.

### Should gynecological pelvic ultrasound be routinely requested as a screening test?

To date, there is no scientific evidence to justify screening for ovarian cancer and endometrial cancer in women at normal risk for these neoplasms. 17 18 19 Therefore, the request for pelvic ultrasound for women with this risk profile who do not present signs and symptoms suggestive of ovarian or uterine diseases does not demonstrate a good cost-benefit relationship. 17 18 19 Among the adverse events of screening in women at normal risk, is the high number of false positives, that is, surgeries and possible associated complications in women who do not have cancer. 20 Note that in symptomatic women, such as those with abnormal perimenopausal uterine bleeding, postmenopausal vaginal bleeding or abdominal discomfort, transvaginal pelvic ultrasound examination is the initial complementary test of choice to assess uterine and ovarian diseases. 11

### How to screen for colorectal cancer?

Colorectal cancer is the third most frequent neoplasm among Brazilian women, with an estimated 20,470 new cases in the 2020-2022 period and an estimated risk of 19.03 cases per 100,000 women. 10 Reducing mortality due to the disease is possible by identifying asymptomatic neoplasms at an early stage through screening. Complementary tests are classified as structural, for example, colonoscopy, and non-structural, for example, fecal occult blood. In cases of positive results in a non-structural test, diagnostic confirmation by colonoscopy is necessary. 21 Screening schemes must be adapted to the resources available in each region. According to the World Health Organization, screening for fecal occult blood from the age of 50 should be performed in countries that can guarantee diagnostic confirmation and treatment. 22 In Brazil, the Ministry of Health considers that people at normal risk for colorectal cancer should be screened from the age of 50 using an yearly fecal occult blood test or colonoscopy with no established frequency. 23 This screening scheme may differ depending on the regional context. The American Cancer Society recommends that screening in patients at normal risk begins at 45 years of age, performed with structural or non-structural tests ( Chart 2 ). 21 The North American Menopause Society (NAMS) considers that from the age of 50, colonoscopy every ten years (if the test result is considered normal) is an appropriate screening regimen for patients at normal risk for colon cancer. 6 Note that suspected cases of colorectal disease should be referred for evaluation by the specialist physician. Screening for patients at high risk for colorectal cancer is outside the scope of this publication.

**Chart 2.** Colorectal cancer screening

Organization	Population screened	Screening options
Ministry of Health 23	Women at normal risk aged between 50 and 75 years	Non-structural tests: - Annual or biennial fecal occult blood Structural tests: - Colonoscopy if positive fecal occult blood
American Cancer Society 21	Women aged 45 to 75 years if life expectancy is greater than 10 years. For women aged 76 to 85, individualization based on patient preferences, life expectancy, and previous screening history	Structural tests: - Colonoscopy every 10 years - Flexible sigmoidoscopy every 5 years - CT virtual colonoscopy every 5 years Non-structural tests: - Annual fecal occult blood - Annual immunochemical fecal test - Fecal DNA every 3 years

**Source:** Wolf et al. 21 and Ministry of Health. 23

### How to screen for risk factors for cardiovascular disease?

After menopause, the beneficial effect of endogenous estrogen on the cardiovascular system is mitigated, and the number of cardiovascular events increases. 24 Screening of risk factors such as diabetes mellitus, arterial hypertension, dyslipidemia, smoking, and obesity is essential for risk stratification and development of treatment plans ( Chart 3 ).

**Chart 3.** Screening of risk factors for cardiovascular disease according to the Ministry of Health

Risk factor	Recommendation	Comments
Dyslipidemia	Screening from 45 years of age in women at high risk for CVD	Screening intervals of every 4-6 years. Age to stop screening not well defined
Obesity	BMI calculation during visits to health services	If BMI changed, plan individual or group behavioral intervention with advice on diet and exercise Waist circumference ≥ 89 cm is considered high and indicative of higher cardiovascular risk
Diabetes mellitus	If there are no risk factors, screen from age 45 with no defined periodicity (possibly every 3-5 years).	Glycosylated hemoglobin (%) Normal: <5.7 Glucose intolerance: 5.7 to 6.4 Diabetes: ≥ 6.5 Fasting blood glucose (mg/dL) Normal: <100 Glucose intolerance: 100 to 125 Diabetes ≥ 126
Arterial hypertension	Screening in adults (>18 years). Frequency not established	Obtain measurements outside the hospital or clinical setting to confirm the diagnosis. > two measurements on two or more visits over a period of one or more weeks
Smoking	Questioning about tobacco use for all adults.	Brief five-step approach (the five A's): 1. Address the use of tobacco; 2. Advise quitting through a clear and personalized message; 3. Assess willingness to quit smoking; 4. Offer assistance to quit; 5. Arrange conditions for patient follow-up and support.

CVD: cardiovascular disease; BMI: body mass index.

**Source:** Ministry of Health. 23

The joint consideration of the therapeutic history and the different individual risk factors is useful for a better determination of the cardiovascular prognosis. Risk calculation tools for the occurrence of events such as myocardial infarction and stroke are available for use. The Brazilian Guideline for Cardiovascular Prevention of the Brazilian Society of Cardiology recommends using the Framingham Global Risk Score as an assessment tool. 25

### How to screen for osteoporosis?

Osteoporosis is often asymptomatic. Its diagnosis through bone densitometry or the documentation of an asymptomatic bone fragility fracture is essential to adopt the appropriate treatment. 26 Bone densitometry should be performed for all women over 65 years of age. Climacteric women under 65 years of age who have some risk factor for low bone mass ( Chart 4 ) should also undergo the examination. 26

**Chart 4.** Risk factors that indicate the need for bone densitometry in climacteric women under 65 years of age

Use of corticosteroids at a dose greater than 5 mg of prednisone/day (or equivalent) for 3 months or more
Low weight
Current smoking
Rheumatoid arthritis
Menopause before age 45
History of bone fragility fracture
Parents with a history of hip fracture
Alcoholism (≥ 3 alcohol units/day)

**Source:** Rosen and Drezner. 26

In cases of doubt regarding the indication of bone densitometry, we recommend the analysis of the FRAX-Brasil using recommendations of the National Osteoporosis Guideline Group (NOGG). 27 The FRAX-Brasil is a computerized algorithm that calculates the probability of occurrence of major osteoporotic fracture and femoral neck fracture in ten years. FRAX and NOGG used together make it possible to select patients who would benefit from performing bone densitometry. Two intervention thresholds are considered based on age-specific fracture probability equivalent to women with a previous fragility fracture. Women classified below the lower limit do not need to undergo bone densitometry, while those above the upper limit are candidates for pharmacological treatment for osteoporosis, regardless of the results of the densitometry test. Those between the lower and upper limits must undergo bone densitometry and subsequently be reclassified according to the FRAX/NOGG. 27 FRAX-Brasil/NOGG is available for use at the following electronic address: https://abrasso.org.br/calculadora/calculadora/ .

Bone fragility fractures occur in the absence of trauma or in the presence of “minor” trauma, often in the thoracolumbar spine, wrist, and hip. 26 They are the most common manifestation of osteoporosis, and may be asymptomatic in up to 70% of cases. As its diagnosis is essential to choose the appropriate therapy and reduce the risk of new fractures, thoracolumbar radiography is recommended for women with the characteristics described in Chart 5 . 28

**Chart 5.** Indication of thoracolumbar radiography for the diagnosis of vertebral fractures in asymptomatic women

Low impact trauma fracture after 50 years
Prolonged treatment with corticosteroids
Loss of historical height a ≥ 4 cm or prospective height b ≥ 2 cm
Age greater than 70 years if spine, femoral neck or total femur BMD T-score ≤ -1.0 c
Age between 65 and 69 years old if the spine, femoral neck or total femur BMD T-score ≤ -1.5 c

BMD: bone densitometry

^a^ Current height compared to the greatest height during adulthood. ^b^ Cumulative height loss measured between medical consultation intervals. ^c^ If bone densitometry is unavailable, radiography may be considered based on age alone.

**Source:** Cosman et al. 28

### How to screen for depression?

The menopausal transition is a window of vulnerability for the development of mood changes and depressive disorders. The risk of presenting symptoms is high during perimenopause, even in women without a personal history of depressive disorder. 29 Identifying women with depressive symptoms is important to adopt the appropriate therapy. Although there are no specific questionnaires for screening for mood disorders in menopausal women, some general screening tools such as the PHQ-9 can be used. This questionnaire is validated for Brazilian Portuguese and a score ≥ 9 identifies individuals at greater risk of having a major depressive episode. 30 Other questionnaires on climacteric symptoms, such as the Menopause Rating Scale, also incorporate questions related to mood and can be used to identify women at risk. 31 It is recommended to make the definitive diagnosis in consultation with a mental health professional.

### How to screen for sexually transmitted infections?

In recent years, there has been an increase in the occurrence of STIs in climacteric and postmenopausal women. The high prevalence of menopausal genitourinary syndrome, which predisposes to bleeding during sexual intercourse, associated with greater accessibility to treatment for male erectile dysfunction, contributes to a greater chance of infection. 32 Behavioral counseling regarding condom use and treatment of the genitourinary syndrome of menopause are important tools to reduce this risk. Screening for STIs should be performed based on data from the clinical history of each patient. 6

### How to screen for thyroid disease?

Thyroid diseases are prevalent in aging women and can increase morbidity and mortality. The Latin American Thyroid Society (LATS) recommends the initial screening of women over 60 years of age for thyroid disease with a thyroid-stimulating hormone (TSH) measurement. 33 The symptoms of thyroid dysfunction are similar to those of hypoestrogenism, therefore, perimenopausal women who experience symptoms such as hot flashes, menstrual irregularity, weight gain, or depression should also have their thyroid function evaluated. 6 33 Screening for thyroid cancer in women at normal risk does not appear to be cost-effective, but clinical evaluation of the thyroid should be performed routinely during physical examination of climacteric women. When there is any change, such as a suspected goiter or nodule, thyroid ultrasound is indicated. 6 33

### What additional tests are necessary before prescribing hormone therapy and during its use?

Menopausal hormone therapy may be indicated to treat vasomotor symptoms associated with hypoestrogenism and the genitourinary syndrome of menopause, in addition to preventing bone loss and reducing the risk of bone fragility fractures. 11 The clinical history and a complete physical examination can rule out the vast majority of contraindications to the use of HT. Suspicious data in the anamnesis should be investigated with complementary exams. Note that the presence of a precursor lesion of breast cancer is a contraindication to the use of HT. Clinical breast examination in asymptomatic women has low sensitivity in the diagnosis of small lesions, which can lead to false negatives. Thus, women who will start HT should have performed a screening mammogram at the maximum of one year earlier. 11

Some complementary exams help in choosing the best route of hormonal administration. Observational studies have shown that transdermal estrogen offers a lower risk of thromboembolic events. 34 The identification of women at greater risk of presenting already formed atheromatous plaques is important to define the best regimen for HT administration. Some international societies recommend the use of cardiovascular risk calculation instruments as an auxiliary tool in the decision on the administration of HT. The North American Menopause Society (NAMS) recommends using the tool developed by the American College of Cardiology available on the internet for use on computers or mobile devices. 35 According to NAMS, women with a risk of less than 10% in 10 years can receive HT, but those with cardiovascular risk between 5% and 10% would benefit more from the transdermal route. 8 The use of estrogen in women with high cardiovascular risk (>10% in 10 years) could destabilize atheromatous plaques and lead to thromboembolic events such as stroke and myocardial infarction. 8 Risk calculation tools use data from anamnesis, physical examination and some laboratory tests, such as total and HDL cholesterol. Therefore, measuring fasting glucose and lipid profile before starting HT is recommended. Even in women at low cardiovascular risk, the transdermal route is more appropriate when triglyceride values are above 400 mg/dL. 8

Ensuring patient safety during medication use is critical, therefore, routine clinical evaluation should be maintained. Cardiovascular risk factors should be reassessed periodically. Chart 3 details the screening schemes proposed by the Brazilian Ministry of Health. Orally administered estrogen can increase serum triglyceride and HDL levels, and decrease LDL levels. The effect is less evident with transdermal administration. An annual assessment of the lipid profile of women using oral HT is recommended. 11 There is no evidence that reducing the breast cancer screening interval to a period of less than one year is beneficial. It is recommended to perform mammographic screening annually. There is no evidence that the discontinuation of hormonal medication for one or two months before the mammogram improves the interpretation of the exam due to a supposed decrease in breast density. 11

Women using systemic continuous combined HT with estrogen and progestagen are expected to have amenorrhea, even though irregular bleeding episodes may occur in the first few months of use. 36 Endometrial evaluation with transvaginal ultrasound and biopsy should be performed if there is persistent bleeding. Women using systemic continuous combined HT with amenorrhea who have a new bleeding episode and those using cyclic HT who have irregular bleeding also need endometrial evaluation. 11 Vaginal low-dose estrogen alone can be used to treat genitourinary menopausal syndrome. In these cases, the use of progestogen is not necessary, but the occurrence of abnormal uterine bleeding requires a prompt complementary endometrial investigation. 37

## Final considerations

Population aging is an established phenomenon in several countries. Aspects related to senility should be a constant reason for consultation with health professionals. The climacteric deserves to be highlighted, as it is accompanied by a series of physiological changes that imply disease prevention and health promotion needs. The medical consultation of climacteric women is an opportunity to screen for chronic diseases and neoplasms. The appropriate workup at this stage of life is one of the initial steps for physicians contributing to the quality of life of their patients.

National Specialized Commission on Climateric of the Brazilian Federation of Gynecology and Obstetrics Associations (FEBRASGO)

President:

Luciano de Melo Pompei

Vice-President:

Lúcia Helena Simões da Costa Paiva

Secretary:

Elizabeth Jeha Nasser

Members:

Ana Lúcia Ribeiro Valadares

Célia Regina da Silva

Eliana Aguiar Petri Nahas

Jaime Kulak Junior

Luiz Francisco Cintra Baccaro

Márcio Alexandre Hipólito Rodrigues

Marco Aurélio Albernaz

Maria Celeste Osório Wender

Maria Célia Mendes

Rita de Cassia de Maio Dardis

Rodolfo Strufaldi

Rogerio Cesar Bocardo

## References

[1] Utian W H Ovarian function, therapy-oriented definition of menopause and climacteric Exp Gerontol 1994 29(3-4):245 51 10.1016/0531-5565(94)90003-5 7925745

[2] Ministério da Saúde. Instituto Sírio-Libanês de Ensino e Pesquisa Protocolos da atenção básica: saúde das mulheres Brasília (DF) Ministério da Saúde 2016

[3] Harlow S D Gass M Hall J E Lobo R Maki P Rebar R W Executive summary of the Stages of Reproductive Aging Workshop + 10: addressing the unfinished agenda of staging reproductive aging J Clin Endocrinol Metab 2012 97 04 1159 68 10.1210/jc.2011-3362 22344196 PMC3319184

[4] Welt C K Ovarian development and failure (menopause) in normal women [Internet] 2020[cited 2020 Jun 21]. Available from:https://www.uptodate.com/contents/ovarian-development-and-failure-menopause-in-normal-women

[5] Federação Brasileira das Associações de Ginecologia e Obstetrícia Insuficiência ovariana prematura São Paulo Febrasgo 2021. (Protocolo Febrasgo-Ginecologia, no. 26/Comissão Nacional Especializada em Ginecologia Endócrina)

[6] NAMS Recommendations for Clinical Care of Midlife Women Working Group Shifren J L Gass M L The North American Menopause Society recommendations for clinical care of midlife women Menopause 2014 21 10 1038 62 10.1097/GME.0000000000000319 25225714

[7] Voda A M Climacteric hot flash Maturitas 1981 3 01 73 90 10.1016/0378-5122(81)90022-0 7253937

[8] Kaunitz A M Manson J E Management of menopausal symptoms Obstet Gynecol 2015 126 04 859 76 10.1097/AOG.0000000000001058 26348174 PMC4594172

[9] Casper R F Clinical manifestations and diagnosis of menopause [Internet] 2020[cited 2020 Jun 21]. Available from:https://www.uptodate.com/contents/clinical-manifestations-and-diagnosis-of-menopause

[10] Ministério da Saúde. Instituto Nacional de Câncer José Alencar Gomes da Silva Estimativa 2020: incidência de câncer no Brasil [Internet] Rio de Janeiro INCA 2019[cited 2020 Jun 1]. Available from:https://www.inca.gov.br/sites/ufu.sti.inca.local/files//media/document//estimativa-2020-incidencia-de-cancer-no-brasil.pdf

[11] Pompei L M Machado R B Wender M C Fernandes C E Consenso Brasileiro de Terapêutica Hormonal da Menopausa – Associação Brasileira de Climatério (Sobrac) São Paulo Leitura Médica 2018

[12] U.S. Preventive Services Task Force Siu A L Screening for breast cancer: U.S. Preventive Services Task Force Recommendation Statement Ann Intern Med 2016 164 04 279 96 10.7326/M15-2886 26757170

[13] Urban L A Chala L F Bauab S P Schaefer M B Santos R P Maranhão N M Breast cancer screening: updated recommendations of the Brazilian College of Radiology and Diagnostic Imaging, Brazilian Breast Disease Society, and Brazilian Federation of Gynecological and Obstetrical Associations Radiol Bras 2017 50 04 244 9 10.1590/0100-3984.2017-0069 28894332 PMC5586515

[14] Committee on Practice Bulletins - Gynecology Practice Bulletin Number 179: breast cancer risk assessment and screening in average-risk women Obstet Gynecol 2017 130 01 e1 e16 10.1097/AOG.0000000000002158 28644335

[15] Ministério da Saúde. Instituto Nacional de Câncer José Alencar Gomes da Silva Diretrizes brasileiras para o rastreamento do câncer do colo do útero [Internet] 2a ed. Rio de Janeiro INCA 2016[cited 2020 Jun 21]. Available from:https://www.inca.gov.br/sites/ufu.sti.inca.local/files//media/document//diretrizesparaorastreamentodocancerdocolodoutero_2016_corrigido.pdf

[16] Federação Brasileira das Associações de Ginecologia e Obstetrícia Rastreio, diagnóstico e tratamento do câncer de colo de útero São Paulo Febrasgo 2017

[17] Menon U Gentry-Maharaj A Burnell M Singh N Ryan A Karpinskyj C Ovarian cancer population screening and mortality after long-term follow-up in the UK Collaborative Trial of Ovarian Cancer Screening (UKCTOCS): a randomised controlled trial Lancet 2021 397(10290):2182 93 10.1016/S0140-6736(21)00731-5 33991479 PMC8192829

[18] Pinsky P F Yu K Kramer B S Black A Buys S S Partridge E Extended mortality results for ovarian cancer screening in the PLCO trial with median 15years follow-up Gynecol Oncol 2016 143 02 270 5 10.1016/j.ygyno.2016.08.334 27615399 PMC5077651

[19] Chen L Berek J S Endometrial carcinoma: clinical features, diagnosis, prognosis, and screening [Internet] 2021[cited 2021 Jul 12]. Available from:https://www.uptodate.com/contents/endometrial-carcinoma-clinical-features-diagnosis-prognosis-and-screening

[20] Committee Opinion No. 716: the role of the obstetrician-gynecologist in the early detection of epithelial ovarian cancer in women at average risk Obstet Gynecol 2017 130 03 e146 9 10.1097/AOG.0000000000002299 28832487

[21] Wolf A M Fontham E T Church T R Flowers C R Guerra C E LaMonte S J Colorectal cancer screening for average-risk adults: 2018 guideline update from the American Cancer Society CA Cancer J Clin 2018 68 04 250 81 10.3322/caac.21457 29846947

[22] World Health Organization Global action plan for the prevention and control of noncommunicable diseases 2013-2020 Geneva WHO 2013

[23] Ministério da Saúde. Secretaria de Atenção à Saúde. Departamento de Atenção Básica Rastreamento [Internet] Brasília (DF) Ministério da Saúde 2010[cited 2020 Jun 23]. (Caderno de Atenção Básica; no. 29). Available from:http://189.28.128.100/dab/docs/publicacoes/cadernos_ab/abcad29.pdf

[24] Lobo R A Hormone-replacement therapy: current thinking Nat Rev Endocrinol 2017 13 04 220 31 10.1038/nrendo.2016.164 27716751

[25] Précoma D B Oliveira G M Simão A F Dutra O P Coelho O R Izar M C Updated Cardiovascular Prevention Guideline of the Brazilian Society of Cardiology – 2019 Arq Bras Cardiol 2019 113 04 787 891 10.5935/abc.20190204 31691761 PMC7020870

[26] Rosen H N Drezner M K Clinical manifestations, diagnosis, and evaluation of osteoporosis in postmenopausal women [Internet] 2020[cited 2020 Jun 21]. Available from:https://www.uptodate.com/contents/clinical-manifestations-diagnosis-and-evaluation-of-osteoporosis-in-postmenopausal-women

[27] Albergaria B H Paula F J The Algorhytm: FRAX Brazil Rev Bras Ginecol Obstet 2019 41 08 467 8 10.1055/s-0039-1695027 31450253 PMC10316822

[28] Cosman F de Beur S J LeBoff M S Lewiecki E M Tanner B Randall S Clinician's guide to prevention and treatment of osteoporosis Osteoporos Int 2014 25 10 2359 81 10.1007/s00198-014-2794-2 25182228 PMC4176573

[29] Maki P M Kornstein S G Joffe H Bromberger J T Freeman E W Athappilly G Guidelines for the evaluation and treatment of perimenopausal depression: summary and recommendations Menopause 2018 25 10 1069 85 10.1097/GME.0000000000001174 30179986

[30] Santos I S Tavares B F Munhoz T N Almeida L S Silva N T Tams B D Sensibilidade e especificidade do Patient Health Questionnaire-9 (PHQ-9) entre adultos da população geral Cad Saúde Pública 2013 29 08 1533 43 10.1590/0102-311X0014461 24005919

[31] Heinemann L A Potthoff P Schneider H P International versions of the Menopause Rating Scale (MRS) Health Qual Life Outcomes 2003 1 28 10.1186/1477-7525-1-28 12914663 PMC183844

[32] Baill I C Castiglioni A Health maintenance in postmenopausal women Am Fam Physician 2017 95 09 561 70 28671391

[33] Brenta G Vaisman M Sgarbi J A Bergoglio L M Andrada N C Bravo P P Clinical practice guidelines for the management of hypothyroidism Arq Bras Endocrinol Metabol 2013 57 04 265 91 10.1590/s0004-27302013000400003 23828433

[34] Mohammed K Abu Dabrh A M Benkhadra K Al Nofal A Carranza Leon B G Prokop L J Oral vs transdermal estrogen therapy and vascular events: a systematic review and meta-analysis J Clin Endocrinol Metab 2015 100 11 4012 20 10.1210/jc.2015-2237 26544651

[35] American College of Cardiology/American Heart Association Task Force on Practice Guidelines Goff D C Jr Lloyd-Jones D M Bennett G Coady S D'Agostino R B Gibbons R 2013 ACC/AHA guideline on the assessment of cardiovascular risk: a report of the American College of Cardiology/American Heart Association Task Force on Practice Guidelines Circulation 2014 129(25 Suppl 2):S49 73 10.1161/01.cir.0000437741.48606.98 24222018

[36] Archer D F Pickar J H Bottiglioni F Bleeding patterns in postmenopausal women taking continuous combined or sequential regimens of conjugated estrogens with medroxyprogesterone acetate. Menopause Study Group Obstet Gynecol 1994 83(5 Pt 1):686 92 8164926

[37] Management of symptomatic vulvovaginal atrophy: 2013 position statement of The North American Menopause Society Menopause 2013 20 09 888 902 10.1097/GME.0b013e3182a122c2 23985562

